# A Hint on Economy

**Published:** 1891-12

**Authors:** 


### A HINT ON ECONOMY.

The lesson which the lower, middle and working classes of our country need to learn is not so much how to get money as how to save it or spend it wisely. Most people can manage the first part of home finance, but it takes a clever person, indeed, to make a proper use of the money when it is earned. Dr. Johnson once said that "without economy none can be rich; and with it few can be poor." And, though his statement cannot be accepted as being absolutely correct, there is still a grain of truth in it.

